# Knowledge and Practice of Burn Management Among Physicians Using Burn Manikin in Ha'il, Kingdom of Saudi Arabia

**DOI:** 10.7759/cureus.36196

**Published:** 2023-03-15

**Authors:** Anas A Fathuldeen, Mohammad A Alduheim, Abdulaziz S Alqahtani, Khalid M Alshammari, Sulaiman S Alsamaan, Abdulrahman H Althagafi, Ziyad H Alanazi

**Affiliations:** 1 Department of Plastic Surgery, College of Medicine, University of Ha'il, Ha'il, SAU; 2 Department of Emergency Medicine, King Khalid Hospital, Ha'il, SAU; 3 Department of Otolaryngology, College of Medicine, University of Ha'il, Ha'il, SAU; 4 Department of Internal Medicine, King Saud Medical City, Riyadh, SAU; 5 Department of Ophthalmology, King Abdulaziz Medical City, Riyadh, SAU; 6 Department of Emergency Medicine, AlHaram Hospital, Madinah, SAU; 7 Department of Internal Medicine, College of Medicine, University of Ha'il, Ha'il, SAU

**Keywords:** intesive care medicine, family medecine, dermatology, healthcare provider, emergency medicine physician, plastic and reconstructive surgery, burn wounds, second degree burn, burn injury, acute burn

## Abstract

Background

Burn is a skin injury that results in the death of the impacted cells. Burn injuries are frequently unintentional and very avoidable. With proper management, the outcome is improved, and the need for surgical intervention is reduced. This article discusses healthcare providers' knowledge and practice of burn first aid and management to highlight the need for the enhanced practice of burn management and first-aid skills.

Objective

This study aims to assess the knowledge and practice of burn injuries management among healthcare workers in different specialties in Hail city.

Methods

A cross-sectional study was conducted via an interviewer-administrated face-to-face questionnaire and video recording of a simulated case of burn injury collected from our skill lab at Hail University and evaluated by a board-certified plastic surgeon.

Result

The study analyzed 119 physicians (mean age = 36.3 years, SD = 6.7) managing burn cases. Of these, 59.7% were male, and 40.3% were female. The mean evaluation score was 7.71 (SD = 2.84). None of the factors studied, including gender (p = 0.353), age (p = 0.970), education level (p = 0.127), specialty (p = 0.871), professional experience (p = 0.118), working sector (p = 0.178), nationality (p = 0.742), or participation in burn management course (p = 0.131), had a significant effect on burn management skills of physicians. However, some groups had higher mean evaluation scores than others. Further research is needed to explore potential reasons for the observed differences in mean evaluation scores among different groups of physicians.

Conclusion

We discovered that most physicians were found to have poor practical knowledge of proper burn management, and most of them had not engaged in a burn first aid training, therefore more courses targeting physicians who may meet burn patients are required.

## Introduction

Acute burn injury is a major health issue worldwide, perhaps a fatal situation that calls for appropriate medical attention. Airway disruptions caused by hot air and smoke inhalation that result in edema are the earliest indications of a life-threatening condition. The most significant long-term and complex pathophysiological repercussions of hypovolemic shock after injury are the most significant subsequent life-threatening dangers [1]. Some burn care facilities have recently achieved significant strides in the successful treatment of acute burns, although they lack the means to do so. Burn injuries are a serious health issue that has an adverse impact on society everywhere. A burn is an injury to the skin or other organic tissue primarily caused by heat or due to radiation, radioactivity, electricity, friction, or contact with chemicals. According to the WHO figures from 2018, about 265,000 deaths are reported each year due to fire alone [2]. Burn-related injuries (BRIs) are fairly prevalent but easily avoidable injuries. First aid in BRIs can significantly affect the outcome and morbidity of these injuries. However, it appears that most healthcare professionals worldwide lack the necessary skills to properly handle burn injuries [3]. Studies have been done to show how much is known about burn first aid therapy (BFAT), which applies to a range of ages and groups. Most of this research revealed that groups, including medical students and healthcare professionals, generally lacked expertise and knowledge of BFAT [4]. According to research [5-9], factors including prior first aid training for burn injuries, educational level, and participation in a course or training for health sector employees are linked to greater levels of knowledge and awareness of burn injury management among medical professionals [1]. The first aid for burns field was not sufficiently understood or perceived by the healthcare professionals, despite their good attitudes toward it. Additionally, Hatan Mortada 2020 reaffirmed the need for an efficient educational program for healthcare professionals to improve their knowledge and comprehension of first aid for burns, which would subsequently boost the success and availability of the best possible care for the patients [10]. Healthcare professionals' knowledge of first aid for burn patients is weak, and there is a high rate of needless traditional medicine and antibiotic use in burn patients. Effective instructional programs for healthcare professionals are crucial [6]. Medical therapies differ depending on the extent of the damage and the severity of the burn. Traditional medicine is frequently used as an alternative to other therapies for minor burns [11]. Patients with various disorders increasingly use herbal medications as a treatment [12]. According to a study done in Saudi Arabia, people with chronic ailments frequently use herbal remedies [13]. Our study aims to assess the clinical knowledge and practice of burn injury management among physicians in Saudi Arabia.

## Materials and methods

Study aim

The main objective of the current study is to assess the level of knowledge of burn injuries management among healthcare workers in the Ha'il region of Saudi Arabia.

Study design

This cross-sectional study was conducted for one year, from November 2021 to November 2022, in Ha'il, the largest city in the northern region of Saudi Arabia.

Study setting

The study was conducted in different healthcare facilities in Ha'il city, Saudi Arabia, including governmental hospitals, private hospitals, and primary healthcare centers. Three governmental hospitals were included in the study: King Salman Specialist Hospital, King Khalid Hospital (the trauma center), and Ha'il General Hospital. Two private hospitals were included in the study: Salamat Hospital and Al Noor Hospital. Primary health care centers were included: Salah Al-Din Health Center, Sababah Health, Al Muntazah Al Gharbi, and Aja Pharmaceutical.

Sampling

By using the Raosoft sample size calculator, the sample size of this study was determined to be no less than 120 participants. Randomly 120 physicians from different specialties (Dermatology, Emergency Medicine, Family Medicine, Intensive Care Medicine, and Surgery) participated in the study.

Inclusion criteria

Both male and female physicians in specific specialties (Surgery, Emergency Medicine, Intensive Care Medicine, Family Medicine, and Dermatology) working in Governmental institutes and the private sector are included in the study.

Data collection

**Figure 1:**
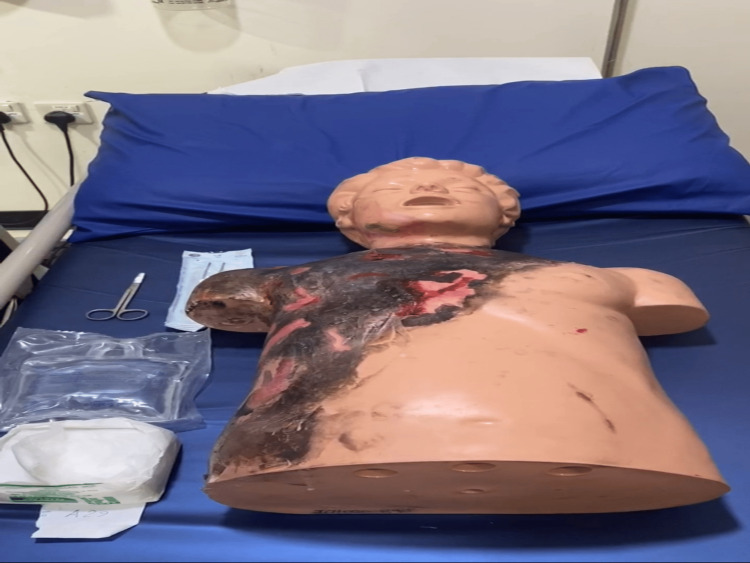
Third degree burn.

**Figure 2:**
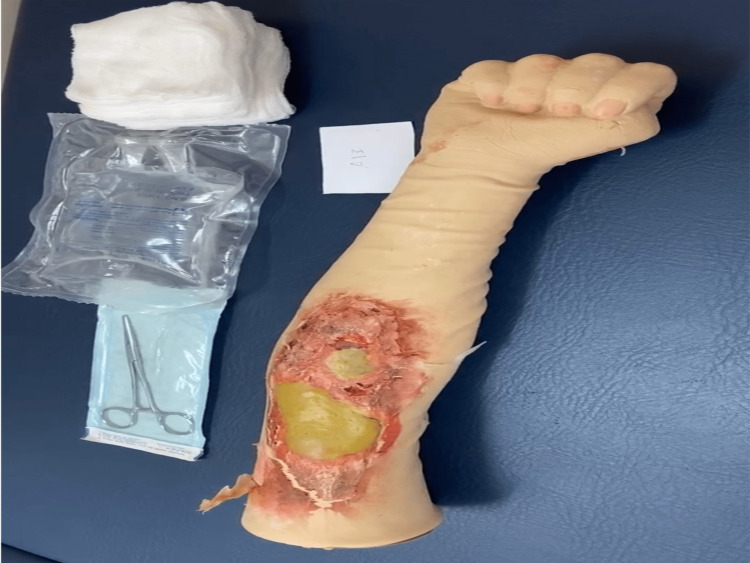
Second degree burn with blister formation.

**Figure 3:**
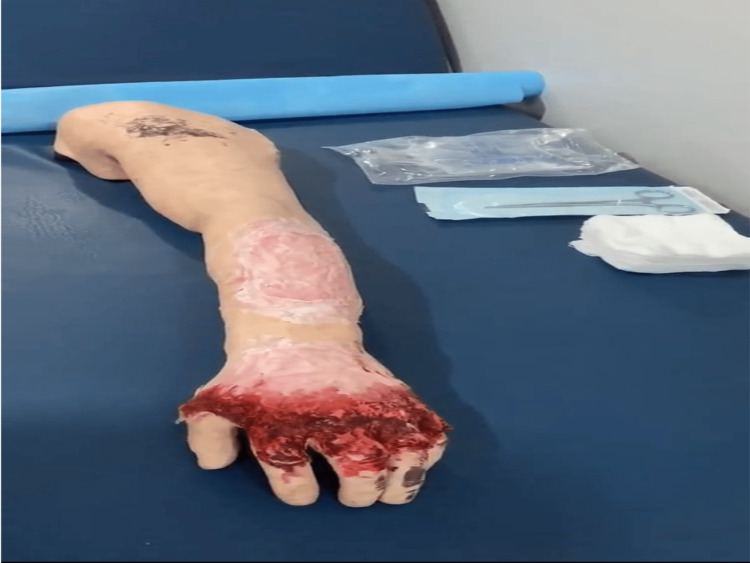
First degree burn.

After explaining the research idea, informed consent was obtained from each participant. Every participant was given a paper containing a burn case scenario with a brief history and the burn manikin used as a real patient; when the participant was ready, we started recording the physician while approaching the mock case without showing the physician's face. There were three manikin cases with varying burn degrees (first, second, and third) (Figures 1-3).

Data collection was divided into two sections. The first section was demographic data which included: case code, age, gender, workplace, nationality, educational level, specialty, experience year, and participation in burn management course. The second section was a video recording of the physician while approaching the case. Every case recorded had a code next to the manikin to link the demographics data with the recording without mentioning the physician's name, workplace, or specialty. After we collected the data, a board-certified plastic surgeon made a Google Form using questions extracted from an article by Alharbi Z et al. (2012) [14] to evaluate each physician. The evaluation had 15 questions, including: Did the doctor do the primary survey (airway, breathing, circulation, disability, and exposure)? Did the doctor do the secondary survey (mechanism of injury and time of injury)? Did the doctor take the temperature? Did the doctor mention an inhalation injury? Did the doctor ask for a referral to the burn center? Did the doctor estimate the total burned surface area correctly? Did the doctor estimate the degree of burn correctly? Did the doctor resuscitate the patient well, depending on the parkland formula? Did the doctor take the vital signs of the patient? Did the doctor take the basic laboratory tests? Did the doctor mention any consultation? Did the doctor do debridement to the patient? Did the doctor mention tetanus and ulcer prophylaxis? Did the doctor ask for analgesia? Did the doctor document the allergies? The minimum score is 0, and the maximum score is 20.

Statistical tools

Both descriptive and inferential statistical analysis of the data was carried out. Simple frequencies and percentages of the sociodemographic characteristics of physicians managing burn cases in the Kingdom of Saudi Arabia and other categorical variables were calculated and tabulated. For evaluation scores and other continuous variables, means and SDs were calculated and reported as measures of central tendency and dispersion, respectively, owing to the relatively normal distribution of the variables assessed by the application and interpretation of Kolmogorov-Smirnov test (p=0.098) (Figure 1). Independent Samples t-test or ANOVA was applied to find a significant difference between means of evaluation scores among different categories of physicians. Statistical significance was established at a p-value of 0.05 or less with a 95% CI. All the statistical calculations were performed using the SPSS Software (by IBM) version 27.0.1.

Ethical consideration

Ethical approval for this study was obtained from the research ethics committee (REC) at the University of Ha'il dated 05/11/2020, and approved by the university president (letter number Nr.16784/5/42 dated 09/11/2020). Confidentiality and privacy were assured for health workers' data.

## Results

The study analyzed 119 physicians satisfying the inclusion criteria who manage burn cases, as shown in Table 1. Of these, 71 (59.7%) were male, and 48 (40.3%) were female. The age of the physicians was grouped into categories, with 38 (31.9%) falling in the 35-39 year age group. Among the physicians, 28 were of Saudi nationality (23.5%), and 91 were non-Saudi (76.5%). Most of the physicians, 107 (89.9%), worked in the government sector, with the remaining 12 (10.1%) in the private sector. Regarding specialization, 55 (46.2%) were specialists, while the rest were consultants or residents. Surgery and family medicine were the most common fields of specialization, with 35 physicians (29.4%) in each category. Only 15 (12.6%) physicians had participated in a burn management course (Table 1).

**Table 1:** Descriptive statistics of different parameters of physicians managing burn cases.

Parameters		Frequency (n = 119)	Percentage (total = 100%)
Gender	Female	48	40.3%
	male	71	59.7%
Age	25-29 y	23	19.3%
	30-34 y	13	10.9%
	35-39 y	38	31.9%
	40-44 y	19	16.0%
	45-50 y	10	8.4%
	Older than 50 y	16	13.4%
Nationality	Non-Saudi	91	76.5%
	Saudi	28	23.5%
Working Sector	Governmental Sector	107	89.9%
	Private Sector	12	10.1%
Educational Level	Consultant	15	12.6%
	Resident	49	41.2%
	Specialist	55	46.2%
Specialty	Dermatology	7	5.9%
	Emergency Medicine	30	25.2%
	Family Medicine	35	29.4%
	Intensive Care Medicine	12	10.1%
	Surgery	35	29.4%
Professional Experience	5-10 years	33	27.7%
	Less than 5 years	31	26.1%
	More than 10 years	55	46.2%
Burn Management Course Participation	Out of 119	15	12.6%

**Figure 4:**
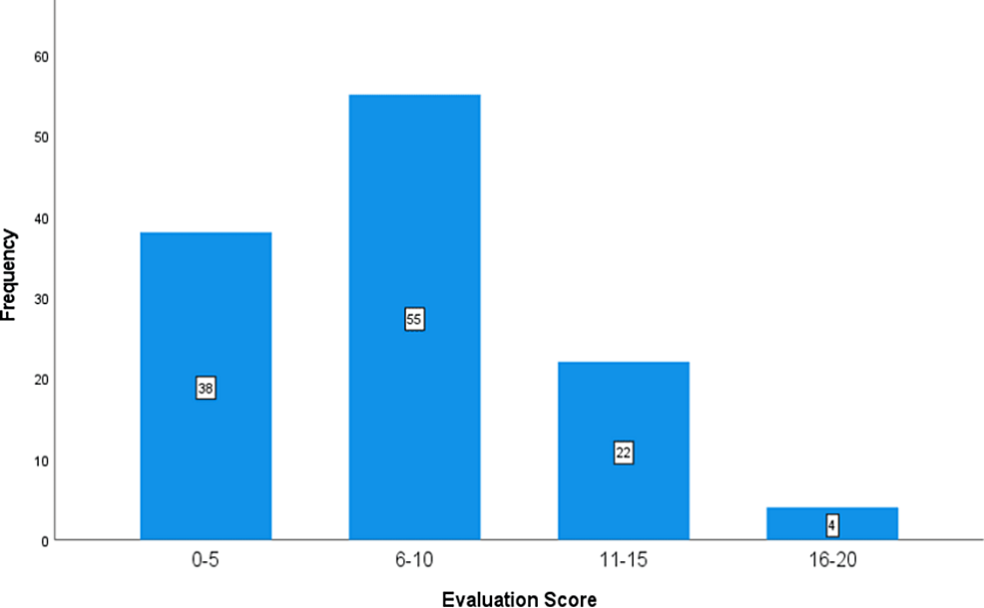
Frequency and evaluation score of physicians in burn management.

The evaluation scores of physicians in burn management were also analyzed, as shown in Table 2 and Figure 4. Out of 119 respondents, 55 (46.2%) scored 6-10 out of 20. The maximum score was 16/20, and the minimum was 0/20 (Table 2).

**Table 2:** Evaluation scores of physicians managing burn cases.

Evaluation Scores (Total = 20)	N (n = 119)	%	Mean	SD	Minimum score	Maximum score
0-5	38	31.9%	7.68	3.87	0/20	16/20
6-10	55	46.2%				
11-15	22	18.5%				
16-20	4	3.4%				

The physicians were evaluated on various parameters, including circulation maintenance, injury mechanism identification, and degree of burn estimation, as shown in Table 3. In the primary survey, 68 (57.1%) physicians maintained circulation in burn management cases, while the distribution of other parameters is shown in Table 3. In the secondary survey, 50 out of 119 (42%) physicians were able to identify injury mechanisms. In other types of surveys, 80 out of 119 (67.2%) physicians correctly estimated the degree of burn, while the rest of the parameters are presented in Table 3.

**Table 3:** Different parameters evaluated by physicians in the management of burn cases.

Survey Type	Parameters Evaluated by Doctors	Number of Doctors (Total = 119)	Percentage (Total = 100%)
Primary Survey	Maintain Airways	66	55.5%
	Maintain Breath	61	51.3%
	Maintain Circulation	68	57.1%
	Check Disability	56	47.1%
	Proper Exposure	65	54.6%
Secondary Survey	Injury Mechanism	50	42.0%
	Injury Times	17	14.3%
Others	Temperature	09	7.6%
	Inhalational Injury Mention	26	21.9%
	Refer Burn Center	53	44.5%
	Correct Estimation of Burn Area	64	53.8%
	Correct Estimation of Burn Degree	80	67.2%
	Resuscitate Patients by PL Formula	63	52.9%
	Vital Signs Taken	32	26.9%
	Order Basic Lab Tests	9	7.6%
	Mention Consultations	55	46.2%
	Debridement of Patients	60	50.4%
	Given Tetanus/UlcersProphylaxis	16	13.4%
	Given Analgesics	58	48.7%
	Ask About Allergy	6	5.0%

Effect of different demographic parameters on the evaluation score of physicians

Upon analyzing the data, it was observed that there were differences in the mean evaluation scores among various groups of physicians. However, these differences were found to be statistically insignificant as p-values were greater than the significance level of 0.05. Therefore, none of the factors being studied significantly affect the burn management skills of physicians. When comparing the mean evaluation scores between males and females, it was found that the mean score of females (8.08) was higher than that of males (7.41). However, this difference was not statistically significant (p = 0.353), as shown in Table 4.

**Table 4:** Effect of gender on evaluation score of physicians managing burn patients.

Gender	N (Total = 119)	Mean	SD	Significance Value
Female	48	8.08	4.094	0.353t
Male	71	7.41	3.721	
Total	119	7.68	3.873	
t = Independent Samples t-test				

Similarly, no significant difference was observed in the mean evaluation scores among physicians of different age groups (p = 0.970, Table 5), educational levels (p = 0.127, Table 6), specialties (p = 0.871, Table 7), or professional experience (p = 0.118, Table 8).

**Table 5:** Effect of age on evaluation score of physicians managing burn patients.

Age	N (Total = 119)	Mean	SD	Significance Value
25-29 y	23	8.17	3.904	0.970f
30-34 y	13	7.46	4.255	
35-39 y	38	7.79	4.021	
40-44 y	19	7.74	3.212	
45-50 y	10	7.20	4.264	
> 50 y	16	7.13	4.129	
Total	119	7.68	3.873	
f = ANOVA				

**Table 6:** Effect of educational level on evaluation score of physicians managing burn patients.

Educational Level	N (Total = 119)	Mean	SD	Significance Value
Consultant	15	8.13	3.091	0.127f
Resident	49	8.41	4.397	
Specialist	55	6.91	3.460	
Total	119	7.68	3.873	
f = ANOVA				

**Table 7:** Effect of specialty on evaluation score of physicians managing burn patients.

Specialty	N (Total = 119 )	Mean	SD	Significance Value
Family Medicine	35	7.37	4.647	0.871f
Dermatology	7	6.86	4.525	
Surgery	35	8.23	3.639	
Emergency Medicine	30	7.67	3.594	
ICU Medicine	12	7.50	2.505	
Total	119	7.68	3.873	
f = ANOVA				

**Table 8:** Effect of professional experience on evaluation score of physicians managing burn patients.

Professional Experience	N (Total = 119)	Mean	SD	Significance Value
5-10 years	33	8.03	3.704	0.118f
Less than 5 years	31	8.65	4.176	
More than 10 years	55	6.93	3.711	
Total	119	7.68	3.873	
f = ANOVA				

Notably, despite the absence of significant differences, some groups had higher mean evaluation scores than others. For instance, physicians aged 25-29 years had the highest mean score (8.17) among all age groups (Table 5), while surgeons had the highest mean score (8.23) among all specialties (Table 6). Similarly, physicians with less than five years of experience had the highest mean score (8.25) among all experience groups (Table 8).

Furthermore, when comparing the mean evaluation scores between physicians who had participated in a burn management course and those who had not, it was found that physicians who had not participated had a higher mean score (8.24) than those who had participated (7.95). However, this difference was also not statistically significant (p = 0.131, Table 9).

**Table 9:** Effect of burn management course on evaluation score of physicians managing burn patients.

Burn Management Course	N (Total = 119)	Mean	SD	Significance Value
No	104	7.88	3.893	0.131t
Yes	15	6.27	3.535	
Total	119	7.68	3.873	
t = Independent Samples t-test				

## Discussion

Numerous studies have shown that proper initial first aid and management of burn injuries significantly lower the number of burn injuries and enhance the quality of life for burn patients [15]. Since practically all patients with burn injuries are handled by trauma center doctors first, their knowledge and professional experience are crucial to the diagnosis, prognosis, treatment, and transfer of those patients to their specialist burns care wards. Burns are frequent and can happen at any time. Acute burn care treatment is a crucial step in the process [16]. A cross-sectional study by Batais MA et al. [17] in Riyadh, Saudi Arabia, on medical and non-medical university students to determine knowledge and practice of burn first aid revealed that 61.8% reported having experienced a burn injury personally or in a clerical setting. Most studies have shown that acute burn injuries management and suitable initial application of first aid can significantly reduce the severity and improve the survival of burn injuries [18]. A total of 54.4% of them claimed to have given or received first aid for burns. According to reported treatments, 81.8% of patients applied water to the damaged region, 72.3% took off clothing or other accessories, and 71 (51.8%) used ice to calm the wound. Another study from Pakistan in Rawalpindi found that the two most often used items after a burn injury were toothpaste (47.5%), followed by cool running water (20.3%), while in Nigeria, water lavage was used in 49 (29.2%) instances, raw eggs in 21 (12.5%), and pap in 16 (9.5%) [19]. Similar findings in two studies by Almutlaq BA et al. (2020) [20] and Al Dhafiri M et al. (2022) [21] conducted in Saudi Arabia found that the majority of participants utilized cold water or honey as post-burn therapy. Although studies have shown that consuming honey does not significantly reduce the amount of burned tissue, it has recently been shown that using honey and silver sulfadiazine together can speed up healing burn wounds [22].

Our study assessed the clinical knowledge and practice of healthcare professionals for the management of patients with acute burn injuries. Its goal was to determine the level of knowledge and practice of burns management among healthcare workers in the Hail region of Saudi Arabia.

Our study showed that dermatologists score less in acute burn management, while surgeons and family medicine physicians were able to score the highest. Our findings differed from those of Lam NN et al.'s findings in that they showed no statistically significant differences between levels of awareness and knowledge of managing burn injuries in relation to participation in burn management courses and work [23], demonstrated that doctors employed by provincial hospitals had much more knowledge than those employed by district hospitals. Doctors who had previously taken training courses had a much greater degree of expertise than the rest. However, fewer people have taken burn first aid courses compared to those who have not, so it is difficult to compare them and say that those who have are less knowledgeable. We found that some participants have also given incorrect referrals; most believe burn cases should be sent to dermatologists rather than plastic surgeons. According to years of experience, the current study demonstrated that there were no statistically significant differences in the level of knowledge and practice of managing burn injuries. Lam NN et al. clarified that work experience did not significantly affect knowledge level, which is consistent with our findings. In contrast to Mortada H et al. study [10], which demonstrated that the mean knowledge score varied significantly across groups of varying ages, the current study showed that there were no statistically significant differences in the level of knowledge and of managing burn injuries according to age groups. In general, the application of scientifically researched and advised treatments for burn first aid is considerably more straightforward, accessible, affordable, and risk-free if done correctly. More training programs aimed at physicians who might encounter burn cases are required. Our study had some limitations. The study was conducted in a single city with limited institutions. There is a possibility that the responses do not represent all healthcare workers in Saudi Arabia. Also, most of the participants had not participated in burn first aid courses. We recommend future research papers that include different institutes from different regions in Saudi Arabia.

## Conclusions

The purpose of this study was to assess the knowledge of burn first aid precisely to provide directed education. Dermatologists score less in acute burn management, while surgeons and family medicine physicians scored the highest. We found that some participants have also given incorrect referrals; most believe burn cases should be sent to dermatologists rather than plastic surgeons. Based on our outcomes, we suggest seeing compulsory basic first aid courses for all physicians who may encounter burn cases on commencement of their employment in the health service coupled with frequent refresher courses.
